# Swelling Dynamics of a DNA-Polymer Hybrid Hydrogel Prepared Using Polyethylene Glycol as a Porogen

**DOI:** 10.3390/gels1020219

**Published:** 2015-11-18

**Authors:** Ming Gao, Kamila Gawel, Bjørn Torger Stokke

**Affiliations:** Biophysics and Medical Technology, Department of Physics, The Norwegian University of Science and Technology, Trondheim NO-7491, Norway; E-Mails: ming.gao@kemi.uu.se (M.G.); kamila.gawel@gmail.com (K.G.)

**Keywords:** DNA competitive displacement, hydrogel swelling, interferometric readout, nanometer resolution, PEG porogen

## Abstract

DNA-polyacrylamide hybrid hydrogels designed with covalent and double-stranded (dsDNA) crosslinks respond to specific single-stranded DNA (ssDNA) probes by adapting new equilibrium swelling volume. The ssDNA probes need to be designed with a base pair sequence that is complementary to one of the strands in a dsDNA supported network junction. This work focuses on tuning the hydrogel swelling kinetics by introducing polyethylene glycol (PEG) as a pore-forming agent. Adding PEG during the preparation of hydrogels, followed by removal after polymerization, has been shown to improve the swelling dynamics of DNA hybrid hydrogels upon specific ssDNA probe recognition. The presence of porogen did not influence the kinetics of osmotic pressure-driven (2-acrylamido-2-methylpropane sulfonic acid)*-co-*acrylamide (AMPSA-*co*-AAm) hydrogels’ swelling, which is in contrast to the DNA-sensitive hydrogels. The difference in the effect of using PEG as a porogen in these two cases is discussed in view of processes leading to the swelling of the gels.

## 1. Introduction

Hydrogels, crosslinked polymer networks imbibed with water, adapt an equilibrium swelling state depending on the molecular parameters of the polymer network and the solution conditions. They adopt swelling states depending on changes in the molecular properties of the network and also altered environmental conditions, such as pH [1,2,3,4], temperature [5,6,7], ionic strength [8,9], electric field [10,11], light [12] and surfactants [13]. Such responsive soft materials tuned to display various physicochemical properties, including swelling, mechanics, permeability and optical properties, have been targeted for various biosensing applications [14,15,16,17,18,19].

**Figure 1:**
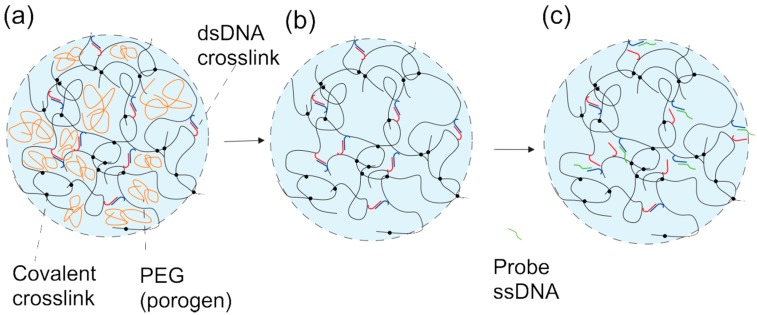
Schematic illustration of the effect of using polyethylene glycol (PEG) as a porogen in the synthesis of DNA hybrid hydrogel and single-stranded DNA competitive displacement of double-stranded, supported crosslinks (**a**–**c**). Hydrogels are prepared in the presence of polyethylene glycol, subsequently removed by washing in buffer, to yield hydrogels with an altered structure (**a**,**b**). DNA hybrid hydrogels prepared in the presence of polyethylene glycol are prepared where the physical crosslinks in the network are made up of double-stranded oligonucleotides with partial complementary bp sequences denoted as sensing (blue) and blocking (red). Swelling response is induced by the oligonucleotide complementary displacement reaction in the presence of an oligonucleotide probe (green), destabilizing the junctions (**b**,**c**), which results in swelling of the hydrogel, determined as the change in the optical length, Δ*l_opt_* using an interferometer.

Changes in the equilibrium swelling volume and dynamics of hydrogels response can be determined using a high resolution interferometric technique. This has previously been applied for the characterization of various types of hydrogels and their swelling responses, including the ionic strength dependence of ionic hydrogels [20], carbohydrates and, in particular, glucose-induced swelling of phenylboronic acid-functionalized hydrogels [21,22,23], nucleotide-sensitive hydrogels [24,25,26], as well as the swelling response associated with the deposition of polymers [27] and surfactants [28]. The swelling of a responsive hydrogel deposited at the tip of an optical fiber is monitored by changes in the optical length of the hydrogel with 2-nm resolution and a sampling frequency at about 1 Hz. In addition, the swelling response of the material itself is rather quick (about 2 s [29]) due to the relatively small size of the hydrogel. The swelling rates, limited by network relaxation, are inversely proportional to the square of the hydrogel’s size [30,31]. Thus, the hydrogel specimen being of only about ~60 μm in radius in the fiber optic-based readout platform yields a rapid swelling/deswelling kinetics that supports a much more rapid readout than methods exploiting larger sizes of the hydrogel specimen. This feature implies that the swelling kinetics of the polymer-DNA hybrid hydrogels in the presence of single-stranded DNA (ssDNA), having a time constant much longer than the 2 s [24,26], is not limited by the swelling response rate of the polymer network, but by other molecular aspects, e.g., associated with the transport and recognition process of the ssDNA probe. Such types of hydrogel are designed to respond through competitive displacement hybridization with the specific ssDNA oligonucleotide integrated in the network (Figure 1).

Two partially-hybridized oligonucleotides, referred to as sensing and blocking oligonucleotides, copolymerized within a hydrogel network, act as physical crosslinks in addition to the covalent ones. The physical crosslinks stabilized by Watson–Crick hydrogen bonds dissociate in the presence of the ssDNA probe with a longer complementary region to the sensing oligonucleotide than the blocking strand. This competitive displacement reaction is a complex process with a range of dynamics depending on the molecular parameters [32,33,34]. The completion of the competitive displacement results in the reduction of the hydrogel crosslink density and concomitant readjustment of the hydrogel swelling volume. The swelling kinetics of these hybrid DNA-polymer hydrogels during such competitive displacement is much slower than that of ionic hydrogels induced by changes in ionic strength [26,29]. Diffusion of ssDNA strands inside polymer-DNA hybrid hydrogel has been shown to be retarded by repeated association and dissociation of the probe with immobile oligonucleotide molecules [35]. Such retarded diffusion may affect the kinetics of swelling towards the equilibrium swelling state of the hydrogel. The repeated association and dissociation processes result in slower equilibration of the hybridization process [35]. The kinetics of polymer-DNA hydrogel biosensor response may nevertheless be tuned through the design of the sensing hydrogel material.

It has been shown that the response rate of polymer-DNA hybrid hydrogel depends on such parameters as: concentration of the physical (double-stranded DNA (dsDNA)) [26] and covalent crosslinks [24], the length of the blocking region within DNA crosslinks and the length of the “toehold” on the ssDNA probe [24] and the presence of a mismatched base on a probe sequence [26]; and does not occur for non-complementary probes [26]. The swelling rate of such DNA-*co-*acrylamide (co-AAm) hybrid hydrogels depends also on the ratio and total concentration of covalent and dsDNA-supported crosslinks. Reduction of the number of base pairs and increasing the length of “toehold” sequences also increase the swelling rate on exposure to a certain concentration of specific ssDNA. The rate of swelling may be even further improved by application of the approaches reported for the preparation of fast-responsive hydrogels that comprise: the synthesis of hydrogels based on comb-type polymers having hydrophobic pendant chains [36] and/or the synthesis of hydrogels with an altered pore size distribution and enhanced porosity [37]. For example, temperature-responsive hydrogels with hydrophobic chains have been shown to shrink more quickly compared to conventional ones when exposed to a temperature stimulus; however, the increase in the collapse rate has not been reported to be associated with an increase in the swelling rate [36]. The approach of enhancing hydrogel porosity to improve the dynamics of both swelling and deswelling has been applied for various stimuli-responsive (pH, temperature, ionic strength) hydrogels. The swelling/deswelling dynamics enhancement is shown to result from a larger mesh size that allows solvent and solute to diffuse more rapidly through the hydrogel. The variety of techniques used to control hydrogel microstructure and porosity has been reviewed [38]. The application of polymers as porogen-forming agents appears to represent a strategy applied by various groups and will be explored here.

The aim of the present work is to explore the possible increase in the swelling kinetics of ssDNA-sensitive hydrogel and to offer more detailed and diversified information about the swelling behavior of porous hydrogels based on the high resolution interferometric technique. Toward this aim, we present how an altered hydrogel pore size distribution induced by adding a porogen during the synthesis facilitates changes in the dynamics of polymer-DNA hybrid hydrogel swelling. The hydrogel pore structure is affected at the stage of hydrogel synthesis by adding inert polyethylene glycol (PEG) during the co-polymerization followed by its removal after the network formation (Figure 1). The approach thus extends the use of PEG as a porogen also to polymer-DNA hybrid hydrogels. The experimental approach includes PEGs with two different molecular weights and introduced at various fractions in the hydrogel preparation procedure and that is subsequently removed by washing. For comparison, the swelling kinetics associated with step changes in ionic strength of the immersing solution for porous AMPSA (2-acrylamido-2-methylpropane sulfonic acid)*-co-*AAm anionic hydrogels is shown to be independent of the variations in the porogen during the synthesis. As swelling of polymer-DNA hybrid hydrogel is affected by the retarded diffusion of the ssDNA probe, this is compared to the kinetics of AMPSA*-co-*AAm hydrogel swelling, which do not possess the DNA-supported crosslink.

## 2. Results and Discussion

### 2.1. AMPSA-co-AAm Hydrogels Synthesized with Various Fractions of Porogens

**Figure 2:**
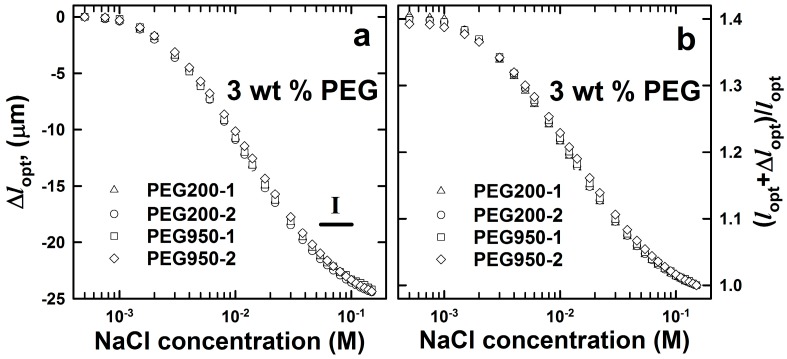
Changes in optical length (**a**) and deswelling ratio (**b**) of (2-acrylamido-2-methylpropane sulfonic acid)*-co-*acrylamide (AMPSA-*co*-AAm) hydrogels synthesized in the presence of pore-forming agents. AMPSA porous hydrogels consisted of 10 wt % AAm, 3 mol % Bis (relative to AAm) and 3 mol % AMPSA (relative to AAm) with 3 wt % PEG (*M*_n_ = 200 g/mol, PEG200, *l*_opt_ = 61.7 µm) and PEG (*M*_n_ = 950~1050 g/mol, PEG950, *l*_opt_ = 62.1 µm) removed after synthesis. All measurements were carried out in aqueous salt solution with the NaCl concentration in the range from 5 × 10^−4^ to 0.15 M and at least two times (labeled as -1 and -2 for each experimental series). Region I depicts the NaCl concentration range selected for displaying the time dependence of Δ*l*_opt_ (Figure 3).

AAm-*co*-AMPSA hydrogels with 3 mol % AMPSA and 3 mol % *N*,*N'*-methylenebisacrylamide (Bis) relative to AAm (10 wt %) were synthesized in the presence of two different molecular weight PEGs (*M*_n_ = 200, 950~1050 g/mol, denoted as: PEG200 and PEG950, respectively) as pore-forming agents representing 3 wt % of the final pre-gel solution. The synthesis of these follows the general scheme outlined in Figure 1 with the adoption of omitting the dsDNA as crosslinks and including a blend of the AMPSA monomer in the AAm pre-gel solution to obtain an anionic hydrogel. The charge and crosslink parameters were chosen to yield a readily-detectable swelling response, in line with the data previously reported for a polycationic hydrogel [29]. The swelling response of synthesized hydrogels to varied ionic strengths was measured using an interferometric technique for hemispherical hydrogels with a radius of about 60 μm attached to an optical fiber. Figure 2 displays the absolute and relative optical length *versus* the ionic strength in the immersion aqueous solution for the hydrogels synthesized in the presence of 3 wt % of PEG200 and PEG950. The relative changes in the optical lengths were obtained by normalizing with respect to the optical length of the cavity measured in 0.15 M NaCl. The swelling curves for ionic strength from 5 × 10^−4^ to 0.15 M showed good reproducibility within each molecular weight of PEG added during the synthesis of the hydrogel. Additionally, the swelling responses for the hydrogels prepared using the two molecular weights of PEG at 3 wt % are identical. The total change of the magnitude of optical length induced by such a difference in concentration of NaCl was about 25 μm. This represents about a 40% change in the optical length of the hydrogel attached to the optical fiber.

**Figure 3:**
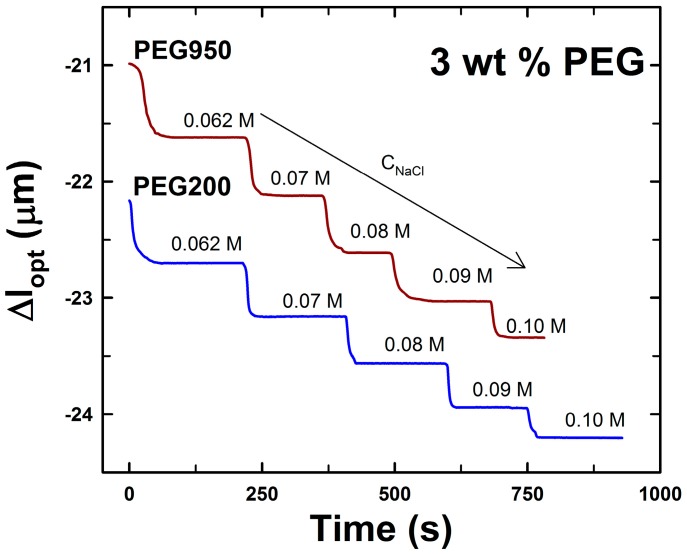
Deswelling kinetics of AMPSA*-co-*AAm hydrogels consisting of 10 wt % AAm, 3 mol % Bis (relative to AAm) and 3 mol % AMPSA (relative to AAm) with 3.33 wt % PEG200 (*l*_opt_ = 61.7 µm) and PEG950 PEG (*l*_opt_ = 62.1 µm) polymers removed right after synthesis. The data are selected for the NaCl concentration range (*c*_NaCl_) from 0.05 to 0.1 M, as indicated (Region I in Figure 2).

Here, we report on the swelling changes of the hemispherical hydrogels attached to the optical fiber as changes in the optical length Δ*l*_opt_ between a reference and the actual state, as deduced from the phase changes of the interference wave. The parameter Δ*l*_opt_ is the primary observable and can be converted to standard measures of changes in hydrogel swelling parameters by applying the strategy as outlined in the following. The primary observable Δ*l*_opt_ can be decomposed into changes due to the altered optical properties of the hydrogels and the change in the physical length,

(1) $$\Delta l_{opt}\;=\;\langle n_{2}\rangle l_{2}-\langle n_{1}\rangle l_{1}\;\approx \langle n_{1}\rangle \Delta l+l_{1}\Delta n$$

where Indices 1 and 2 represent the two states to be compared, e.g., at two different NaCl concentrations in the case of the AAm-*co*-AMPSA hydrogels, and:

(2) $$\langle n_{i}\rangle =l_{i}^{-1}\int_{0}^{l_{i}}n_{i}(l)dl\;,\;i\text{ = 1,2}$$

are the refractive indices averaged along the optical pathway of the two states, indicated by the indices. For most cases, the change in the physical length is the larger of these contributions, e.g., for cyclodextrin-induced swelling changes of cholesterol-modified pullulan hydrogels, we estimated that the relative changes due to altered optical properties were *l*_1_Δ*n/l*_opt_ up to 4 × 10^−4^ at a cyclodextrin concentration of 3 mM [39]. The finding that this was much less than Δ*l*_opt_/*l*_opt_ led to the conclusion that changes in the physical length of the hydrogel along the optical axis are by far the dominating contribution (Equation (1)). The optical length within the hydrogel, *l*_opt_, is determined from the frequency difference between two neighboring frequency peaks in the interference spectrum [29]. The experimentally-determined Δ*l*_opt_/*l*_opt_ can be converted to relative volume changes, *V*/*V*_0_, using the relation *V*/*V*_0_ ~ [(Δ*l*_opt_ + *l*_opt_)/*l*_opt_]^2.6^. The power law coefficient of 2.6, different from 3, arises due to the constraining of the hydrogel at the end of the optical fiber and is deduced from finite element analysis of such a hydrogel [40]. Data converted following this outline provide information similar to that by analyzing trends in the changes of the optical length directly.

Figure 3 presents deswelling kinetics for the two hydrogels PEG200 and PEG950 for the step changes in NaCl concentration from 5.4 × 10^−2^ to 0.1 M (concentration Range I depicted in Figure 2). The data indicate that deswelling kinetics did not depend on the molecular weight of the PEG added during the synthesis of the AMPSA hydrogels. The apparent equilibration time constants τ_1/2_ were found to be in the range of 10 to 15 s. These time constant values correspond to kinetic constants estimated for ionic strength-induced *N*-(3-dimethylaminopropyl)-*co*-AAm hydrogel deswelling in the ionic strength range from 0.03 to 0.074 M [29].

**Figure 4:**
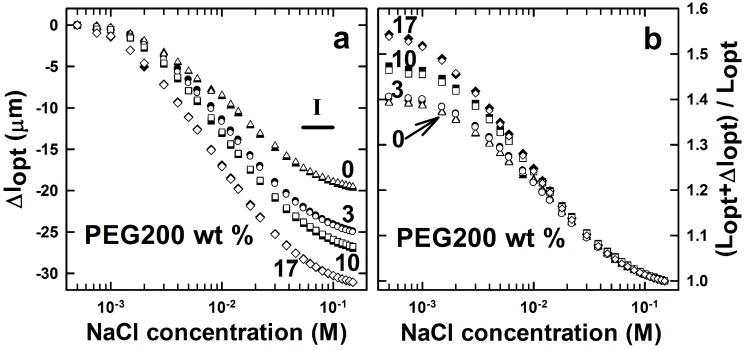
Changes in the optical length (**a**) and deswelling ratio (**b**) of AMPSA*-co-*AAm hydrogels consisting of 10 wt % AAm, 3 mol % Bis (relative to AAm) and 3 mol % AMPSA (relative to AAm) and with 0 (AMPSAGel0, *l*_opt_ = 50.3 µm), 3 wt % (AMPSAGel3, *l*_opt_ = 61.7 µm), 10 wt % (AMPSAGel10, *l*_opt_ = 57.6 µm) and 17 wt % (AMPSAGel17, *l*_opt_ = 57.2 µm) of PEG200 porogen removed right after synthesis. All measurements were carried out in NaCl concentrations ranging from 5 × 10^−4^ to 0.15 M and at least two times (open and filled symbols for each experimental series). Region I depicts the concentration range of NaCl selected for displaying the time dependence of Δ*l*_opt_ (Figure 5).

Whereas the molecular weight of PEG polymer did not influence either the swelling capacity or the kinetics of AMPSA-*co*-AAm hydrogels, the weight fraction of the pore-forming agent was shown to affect the swelling capacity (Figure 4).

Hydrogels were synthesized with four different fractions of PEG200 in the pre-gel solution: 0, 3, 10 and 17 wt %. These data (Figure 4) show that the ionic strength-induced swelling capacity of the AMPSA-*co*-AAm hydrogels was found to increase with the increasing fraction of the PEG200 included during the synthesis. This is consistent with the results reported by the others [41]. The presence of inert polymer, such as PEG, in the pre-gel solution during polymerization was shown to influence hydrogel network arrangement and result in changes in the hydrogel pore size distribution [42].

**Figure 5:**
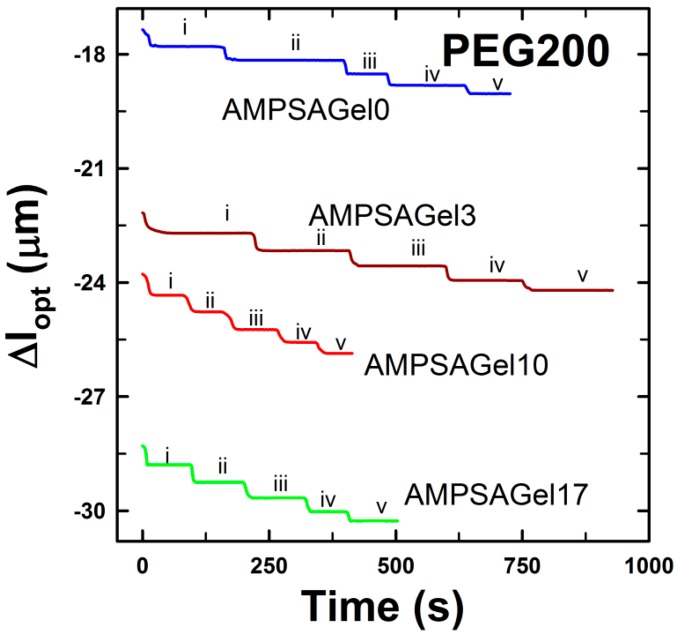
Deswelling kinetics of AMPSA*-co-*AAm hydrogels. The AMPSA*-co-*AAM hydrogels consisted of 10 wt % AAM, 3 mol % Bis (relative to AAm) and 3 mol % AMPSA (relative to AAm) and 0 (AMPSAGel0, *l*_opt_ = 50.3 µm), 3 wt % (AMPSAGel3, *l*_opt_ = 61.7 µm), 10 wt % (AMPSAGel10, *l*_opt_ = 57.6 µm) and 17 wt % (AMPSAGel17, *l*_opt_ = 57.2 µm) of PEG200 porogen removed after synthesis, respectively. The data are selected from Region I in Figure 4, and the actual NaCl concentrations depicted are 0.062 M (i), 0.07 M (ii), 0.08 M (iii), 0.09 M (iv) and 0.1 M (v).

The experimental procedure applied for the synthesis of AMPSA*-co-*AAm and DNA-*g*-AAm hydrogels assures a constant fraction of polymer for the hydrogels within the same compositional type (AMPSA- or DNA-type hydrogel series). This indicates that the total pore volume of the hydrogels is unchanged when including PEG in the mixed solvent during synthesis of the hydrogel, and the observed effects are suggested to originate from changes in the pore size distribution due to the various PEG contents present during the synthesis. The altered pore size distribution manifests itself in an increased hydrogel swelling ratio and swelling deswelling kinetics compared to hydrogels synthesized without PEG in the solvent [43]. The faster swelling/deswelling kinetics is believed to occur due to facilitated solvent and solute transport inside the hydrogel with an altered pore size distribution [30,43]. However, the kinetics of the ionic strength-induced deswelling of AMPSA-*co*-AAm hydrogels was comparable for all gels and independent of PEG content (Figure 5). The apparent deswelling time constants were at the order of a few seconds. For the hydrogel sizes used here, a hemispherical geometry with a radius of about 60 μm, it is reported that the kinetics of swelling with time constants of a few seconds corresponds to the network diffusion limit [29]. This indicates that variations in hydrogel structure induced by including PEG as the porogen do not influence the diffusivity of the hydrogel network.

### 2.2. DNA-g-AAm Hydrogels with Altered Pore Size Distribution

DNA-*g*-AAm hybrid hydrogels crosslinked with 0.6 mol % of covalent (Bis) and 0.4 mol % of physical DNA (sensing-blocking (S-B) oligonucleotide complex) crosslinks were synthesized in the presence of different fractions (0 (DNAGel0), 10 (DNAGel10), 17 (DNAGel17) wt %) of PEG200 inert polymer in the pre-gel solution. The base pair sequence of the S oligonucleotide was chosen at random and the B oligonucleotide with a 12-base pair complementarity to the S oligonucleotide (Table 1). The probe oligonucleotide was chosen to have a six base pair longer complementary sequence than the blocking oligonucleotide (six base pairs in the so-called “toe-hold” region beyond the 12 base pairs in the complementary region) (Table 1). Similar to that for the AMPSA-*co*-AAm hydrogels, the presence of PEG200 during the polymerization and subsequent removal was assumed to affect the experimentally-determined swelling dynamics by altering the structure and pore size characteristics of the DNA-*g*-AAm hybrid hydrogels. The swelling response of the prepared DNA-*g*-AAm hydrogels was explored in the presence of probe oligonucleotide P comprising 18 base pairs complementary to the sensing strand (S). The competitive displacement reaction between the S-B hybridized pair and probe P dissociates the S-B-supported crosslinks and leaves an S-P complex immobilized to the AAm network through the grafted S oligonucleotide. The reduction of the crosslink density associated with this competitive displacement reaction affects the swelling of the hydrogel. A schematic illustration of the response of a DNA hybrid hydrogel with an altered pore size distribution as synthesized at the end of the optical fiber is given in Figure 1.

**Table 1:** Sequence of oligonucleotides used for double-stranded (dsDNA) supported junctions in the DNA-*g*-AAm hydrogels with an altered pore size distribution. The oligonucleotide base pair (bp) sequence is depicted for the sensing (S, blue in Figure 1) and blocking (B, red in Figure 1) oligonucleotides of the dsDNA-supported junctions and probe oligonucleotide (P, green in Figure 1) used for assessing the hydrogel swelling response. The underlined parts of the bp sequences denote the complementary region.

Oligonucleotide	Base Pair Sequence
Sensing (blue)	5′-CTG ATC TAA GTA ACT ACT AG-3′
Blocking (red)	5′-CTC AGT CAC TAG TAG TTA CT-3′
Probe (green)	5′-CTA GTA GTT ACT TAG ATC-3′

**Figure 6:**
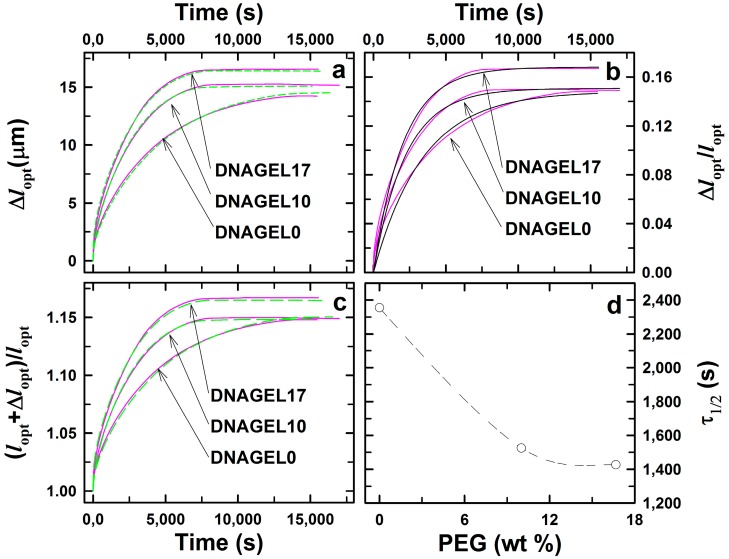
Optical length changes (Δ*l*_opt_) (**a**) and optical length changes relative to the optical length at reference state ((*l*_opt_ + Δ*l*_opt_)/*l*_opt_ (**c**) of DNA hybrid hydrogels with an altered pore size distribution upon ssDNA probe recognition. Fits of first order swelling kinetics (black lines) to relative optical length changes (Δ*l*_opt_/*l*_opt_, pink lines) (**b**) were the basis for the estimation of apparent equilibration time constants (τ_1/2_ = ln2/k) (**d**). DNA hybrid porous hydrogels consisted of 10 wt % AAm, 0.6 mol % Bis (relative to AAm) and 0.4 mol % S-B double-stranded oligonucleotides (relative to AAm) synthesized in the presence of 0 (DNAGel0, *l*_opt_ = 96.2 µm), 10 (DNAGel10, *l*_opt_ = 101.8 µm) and 17 wt % (DNAGel17, *l*_opt_ = 99.1 µm) of PEG200. All measurements were carried out with a probe nucleotide concentration of 20 µM in buffer solution at room temperature and at least two times (red and green lines for each experimental series).

Figure 6 presents the swelling kinetics of DNA-*g*-AAm hydrogels when probe nucleotide P is added to the aqueous buffer (160 mM ionic strength, pH 7), yielding the probe concentration of 20 µM. The swelling process was observed to be faster for the DNA-*g*-AAm hydrogels for increasing PEG content in the pre-gel solution. Figure 6d compares the swelling equilibration time constants for samples DNAGel0, DNAGel10 and DNAGel17. The hydrogel DNAGel0 synthesized without the addition of pore-forming agent showed the longest equilibration time constant τ_1/2_ = 2360 s. The constant decreased to 1530 and 1430 s for the DNAGel10 and DNAGel17 samples, respectively. Equilibration time constants measured for DNA-*g*-AAm hydrogels were about two orders of magnitude higher than the time constants for ionic strength-induced AMPSA*-co-*AAm deswelling, found to be in the range of 10 to 15 s. This remarkable difference in kinetics results from the physico-chemical nature of the two processes. Diffusion of probe oligonucleotides inside the hydrogel network with grafted DNA strands is reported to be interrupted by subsequent association and dissociation processes of the probe with complementary base pairs with immobile DNA strands [35]. As a result, the diffusion of the probe is much slower than in hydrogels without complementary oligonucleotides grafted to the network. Changes in the DNA-*g*-AAm hydrogel porous structure induced by PEG may affect oligonucleotide transport by various mechanisms: by increasing hydrogel permeability and by decreasing the probability of probe-immobile strand interaction. It is also conceivable that an altered porous structure can affect the rate of the competitive displacement hybridization reaction. Steric constraints imposed by the proximity of the acrylamide backbone that are a possible obstacle for the displacement process may be affected by the change of the hydrogel pore distribution. Nevertheless, further experimental evidence would be required to assess the relative importance of the two described mechanisms on DNA-*g*-AAm hydrogels’ swelling dynamics.

## 3. Conclusions

In this work, we present how the kinetics of the swelling response of DNA-*g*-AAm hydrogel to an ssDNA probe may be facilitated by introducing polyethylene glycol as a means to affect the DNA-*g*-AAm hybrid hydrogel porous structure. Swelling of DNA-*g*-AAm hydrogel in the presence of a complementary nucleotide probe occurs due to the destabilization of physical DNA crosslinks [26]. Swelling kinetics is governed by the kinetics of the competitive displacement reaction between the probe and DNA physical crosslinks, and the rate of this process is dependent on the diffusion rate of the probe within the hydrogel matrix. The ssDNA diffusion within the polymer-DNA hybrid hydrogel is slowed down by repeated association and dissociation of the probe with immobile DNA strands, which is known as “retarded diffusion” [35]. Quantitative modelling of the swelling kinetics, including also additional experimental approaches for the determination of relevant parameters, including these various identified molecular processes and network relaxation, is a topic for further exploration. Altering the hydrogel pore size distribution resulted in enhanced permeability of the material and a decreased probability of multiple probe-immobile DNA interactions, which led to faster hydrogel equilibration. The equilibration was faster the higher the weight fraction of PEG in the pre-gel solution was. This suggests that the hydrogel pore size distribution was showing an increased fraction of larger pores for higher PEG content. In contrast to the “retarded diffusion” limited swelling of DNA-*g*-AAm hydrogel, osmotic pressure-driven AMPSA-*co*-AAm hydrogel deswelling has not been affected by the weight fraction of porogen. The finding suggests that this hydrogel network diffusivity, which is a hydrogel deswelling limiting factor, is not strongly affected by changes in the pore size distribution of the network.

## 4. Experimental Section

DNA and AMPSA-*co*-acrylamide-bis-acrylamide hydrogels were synthesized at the end of optical fibers functionalized with 3-(trimethoxysilyl) propyl methacrylate (>98%, Aldrich, St Louis, MO, USA) according to the previously-described procedure [26]. Briefly, the DNA hybrid hydrogels (DNA-*g*-AAm) were synthesized using acrylamide (AAm, 99%, Sigma, St Louis, MO, USA), (*N*,*N*-methylene-bisacrylamide, (Bis, 99%+, Acros organics, Geel, Belgium) as the covalent crosslinker, hybridized oligonucleotides functionalized at the 5′ end (Acrydite, Integrated DNA Technologies, Coralville, IA, USA) acting as reversible physical crosslinks and in the presence of pore-forming agent polyethylene glycol (PEG, *M*_n_ = 200 g/mol, Aldrich), denoted as PEG200. The functionalized oligonucleotides contain an acrylic phosphoramidite as a 5′-modification of the oligonucleotide and can be incorporated into polyacrylamide gels upon polymerization. The incorporated oligonucleotides are connected with a 6-carbon spacer to the AAm network (Integrated DNA Technologies) [44]. AMPSA*-co-*AAm hydrogels were synthesized using 2-acrylamido-2-methyl-1-propane-sulfonic acid (AMPSA, 99%, Aldrich) as a co-monomer, 3 mol % Bis relative to AAm and in the presence of two PEG polymers with different molecular weights, *M*_n_ = 200 (PEG200) and 950~1050 g/mol (PEG950) (Aldrich), and different contents (0, 3, 10, 17 wt %). The hydrogels prepared with 0, 3, 10 and 17 wt % of PEG200 during the synthesis are denoted as AMPSAGel0, AMPSAGel3, AMPSAGel10 and AMPSAGel17, respectively.

The DNA-*g*-AAm hybrid hydrogels were synthesized using sensing oligonucleotide S with the nucleotide sequence chosen at random. The blocking oligonucleotide B was designed with 12 base pairs complementary to S. The probe oligonucleotide was chosen to have a 6 base pair longer complementary sequence than the blocking oligonucleotide (6 base pairs in the so-called “toe-hold” region beyond the 12 base pairs in the complementary region). The sequences of the oligonucleotides used were as follows: sensing S: 5′-CTGATCTAAGTAACTACTAG-3′, blocking B: 5′-CTCAGTCACTAGTAGTTACT-3′), probing P: 5′-CTAGTAGTTACTTAGATC-3′. Acrydite oligonucleotides were dissolved in AAm (10 wt %), 0.6 mol % Bis relative to AAm and PEG *M*_n_ = 200 g/mol (0, 10, 17 wt %) solution prepared in an aqueous buffer (pH 7.0, 10 mM Tris (99.8% Sigma Aldrich, St Louis, MO, USA), 1 mM EDTA (Sigma Aldrich, 99%), 150 mM NaCl (Sds, 99%), yielding a total ionic strength of about 160 mM). Hydrogels prepared using 0, 10 and 17 wt % of PEG200 are denoted as DNAGel0, DNAGel10 and DNAGel17, respectively. Appropriate volumes of the solutions to yield a 0.4 mol % nucleotide concentration of S and B were mixed together at least 3 h (room temperature) before the synthesis to allow S-B hybridization prior to the cross-linking co-polymerization.

**Figure 7:**
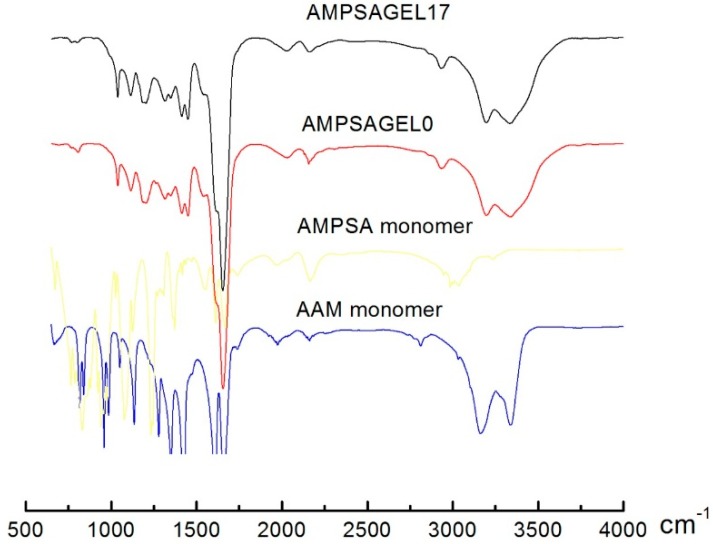
FT-IR of AAm monomer, AMPSA monomer, freezing-dried AMPSAGel0 and AMPSAGel17.

Pre-gel solutions were deposited at the end of a functionalized optical fiber. UV-initiated polymerization was carried out for 6 min using Dymax Bluewave as the UV light source and a photo-initiator (hydroxycyclohexyl phenyl ketone, 99%, Aldrich). The pre-gel deposition at the end of the fiber and polymerization were carried out in a squalane oil (99%, Aldrich) to prevent water evaporation from the hydrogels [26]. Following the photo-polymerization, the fibers with attached hydrogels were protected with glass tubes with an inner diameter of about 1 mm. Hydrogels attached to the optical fiber were immersed in ultrapure water (AMSPSA hydrogels) or PBS buffer (DNA hydrogels) for enough time (24 h), changing the solutions from time to time. No further changes in the interferometer phase signal were employed as a guideline for reaching the equilibrium state of the hydrogel before proceeding with the characterization of the swelling response. It was confirmed by FT-IR (Figure 7) that such a washing and equilibration procedure yielded hydrogels without detectable signatures of PEG. The DNA-grafted hydrogels were pre-equilibrated at 23 °C in buffer solution prior to exposure to probe P buffered solution. The experiments were carried out as follows: the fibers/tubes were taken out of the buffer solution and immersed in 500 µL of the 20 µM oligonucleotide probe P in the buffered solutions thermally equilibrated at 23 °C.

The AMPSA*-co-*AAm hydrogels with PEG content of 0, 3, 10, 17 wt % were synthesized and washed similar to the DNA-*g*-AAm hydrogels. The AMPSAGels were characterized by Fourier transform infrared spectra (FT-IR, PerkinElmer spectrum One FT-IR spectrometer) by depositing a droplet of pre-gel solution on the glass substrate, followed by UV polymerization for 3 min, and washed and stored in ultrapure water for 3 days with changing of the water from time to time. The samples were freeze-dried before FT-IR. The FT-IR spectra indicate a hydrogel structure comprising the monomers in the pre-gel solution (Figure 7). No specific curve of PEG (2870 cm^−1^) observed by FT-IR proved the complete removal of porogen in AMPSA hydrogels.

A typical FT-IR curve of AMPSA-*co*-AAm gels and their monomers is shown in Figure 7. The stretching vibration peaks at 3432 and 1647 cm^−1^ were respectively for amino and amidic carbonyl groups of AAm monomer and AMPSA gels. The peaks around 1039 and 1183 cm^−1^ were the characteristic peaks of the asymmetric and symmetric bands of sulfonate groups in the AMPSA unit, which also appeared for AMPSAGEL0 and AMPSAGEL17. The disappearance of the peak around 1600 cm^−^^1^ (vinyl groups) further confirmed the formation of AMPSA-*co*-AAm gels. The FT-IR spectra indicated a hydrogel structure comprising the monomers in the pre-gel solution.

The AMPSA*-co-*AAm hydrogels were immersed and equilibrated in 40 mL of 0.5 mM NaCl aq solution prior to the determination of the swelling properties. Hydrogel swelling of the AMPSA*-co-*AAm hydrogels was determined by stepwise adjusting the NaCl concentration in the immersing bath by pipetting aliquots of 2 M NaCl into the immersing solution under constant mixing.

Changes in the optical length of the hydrogels (Δ*l*_opt_) were determined using the high resolution interferometric readout technique described in detail previously [29]. The experimentally-determined phase change of the interference wave was used as the basis for the determination of Δ*l*_opt_ due to its superior resolution compared to data extracted from the amplitude of the interference wave. The measurement was carried out until the hydrogel reached equilibrium, as manifested by the constant phase on the interferometric readout. Changes in the optical length of the hydrogel, Δ*l*_opt_, relative to the overall optical length of hydrogels *l*_opt_, Δ*l*_opt_/*l*_opt_ were employed as a measure of the hydrogel swelling behavior. Parameter Δ*l*_opt_ is determined relative to *l*_opt_ selected as the reference state for each experimental series. The changes of the swelling ratio of the hydrogels following exposure to altered salt concentration or ssDNA probe (one concentration) were followed as a function of time. The Δ*l*_opt_/*l*_opt_ was observed to change exponentially with time. An exponential decay was observed in the case of deswelling hydrogels exponentially increased to a new plateau maximum for the swelling gels. The kinetics of swelling was analyzed based on an apparent first order rate process of Δ*l*_opt_/*l*_opt_. The swelling/deswelling kinetics were quantitatively represented by an apparent equilibration time constant (τ_1/2_ = ln2/*k*) by fitting Δ*l*_opt_(*t*)/*l*_opt_ = e^−*kt*^ to the experimental data.

## References

[1] Ricka J., Tanaka T. (1984). Swelling of ionic gels: Quantitative performance of the donnan theory. Macromolecules.

[2] Brannon-Peppas L., Peppas N.A. (1990). Dynamic and equilibrium swelling behaviour of pH-sensitive hydrogels containing 2-hydroxyethyl methacrylate. Biomaterials.

[3] Brannon-Peppas L., Peppas N.A. (1991). Time-dependent response of ionic polymer networks to pH and ionic strength changes. Int. J. Pharm..

[4] Beebe D.J., Moore J.S., Bauer J.M., Yu Q., Liu R.H., Devadoss C., Jo B.-H. (2000). Functional hydrogel structures for autonomous flow control inside microfluidic channels. Nature.

[5] Miyata T., Nakamae K., Hoffman A.S., Kanzaki Y. (1994). Stimuli-sensitivities of hydrogels containing phosphate groups. Macromol. Chem. Phys..

[6] Yoshida R., Uchida K., Kaneko Y., Sakai K., Kikuchi A., Sakurai Y., Okano T. (1995). Comb-type grafted hydrogels with rapid deswelling response to temperature changes. Nature.

[7] Lee W.F., Yuan W.Y. (2000). Thermoreversible hydrogels X: Synthesis and swelling behavior of the (*N*-isopropylacrylamide-*co*-sodium 2-acrylamido-2-methylpropyl sulfonate) copolymeric hydrogels. J. Appl. Polym. Sci..

[8] Schröder U.P., Oppermann W. (1996). Properties of polyelectrolyte gels. Physical Properties of Polymeric Gels.

[9] Park T.G., Hoffman A.S. (1993). Sodium chloride-induced phase transition in nonionic poly(*N*-isopropylacrylamide) gel. Macromolecules.

[10] Grimshaw P.E., Nussbaum J.H., Grodzinsky A.J., Yarmush M.L. (1990). Kinetics of electrically and chemically induced swelling in polyelectrolyte gels. J. Chem. Phys..

[11] Sun S., Mak A.F.T. (2001). The dynamical response of a hydrogel fiber to electrochemical stimulation. J. Polym. Sci. B Polym. Phys..

[12] Mamada A., Tanaka T., Kungwatchakun D., Irie M. (1990). Photoinduced phase transition of gels. Macromolecules.

[13] Eeckman F., Moes A.J., Amighi K. (2003). Surfactant induced drug delivery based on the use of thermosensitive polymers. J. Controll. Release.

[14] Holtz J.H., Asher S.A. (1997). Polymerized colloidal crystal hydrogel films as intelligent chemical sensing materials. Nature.

[15] Miyata T., Asami N., Uragami T. (1999). A reversibly antigen-responsive hydrogel. Nature.

[16] Alexeev V.L., Sharma A.C., Goponenko A.V., Das S., Lednev I.K., Wilcox C.S., Finegold D.N., Asher S.A. (2003). High ionic strength glucose-sensing photonic crystal. Anal. Chem..

[17] Yang Z.M., Gu H.W., Fu D.G., Gao P., Lam J.K., Xu B. (2004). Enzymatic formation of supramolecular hydrogels. Adv. Mater..

[18] Kim H., Cohen R.E., Hammond P.T., Irvine D.J. (2006). Live lymphocyte arrays for biosensing. Adv. Funct. Mater..

[19] Yang Z., Ho P.-L., Liang G., Chow K.H., Wang Q., Cao Y., Guo Z., Xu B. (2007). Using β-lactamase to trigger supramolecular hydrogelation. J. Am. Chem. Soc..

[20] Tierney S., Sletmoen M., Skjak-Braek G., Stokke B.T. (2010). Interferometric characterization of swelling of covalently crosslinked alginate gel and changes associated with polymer impregnation. Carbohydr. Polym..

[21] Tierney S., Falch B.M.H., Hjelme D.R., Stokke B.T. (2009). Determination of glucose levels using a functionalized hydrogel-optical fiber biosensor: Toward continuous monitoring of blood glucose *in vivo*. Anal. Chem..

[22] Tierney S., Volden S., Stokke B.T. (2009). Glucose sensors based on a responsive gel incorporated as a fabry-perot cavity on a fiber-optic readout platform. Biosens. Bioelectron..

[23] Skjaervold N.K., Solligard E., Hjelme D.R., Aadahl P. (2011). Continuous measurement of blood glucose validation of a new intravascular sensor. Anesthesiology.

[24] Gao M., Gawel K., Stokke B.T. (2011). Toehold of dsdna exchange affects the hydrogel swelling kinetics of a polymer-dsDNA hybrid hydrogel. Soft Matter.

[25] Gawel K., Stokke B.T. (2011). Logic swelling response of DNA-polymer hybrid hydrogel. Soft Matter.

[26] Tierney S., Stokke B.T. (2009). Development of an oligonucleotide functionalized hydrogel integrated on a high resolution interferometric readout platform as a label-free macromolecule sensing device. Biomacromolecules.

[27] Gawel K., Gao M., Stokke B.T. (2012). Impregnation of weakly charged anionic microhydrogels with cationic polyelectrolytes and their swelling properties monitored by a high resolution interferometric technique. Transformation from a polyelectrolyte to polyampholyte hydrogel. Eur. Polym. J..

[28] Gao M., Gawel K., Stokke B.T. (2013). High resolution interferometry as a tool for characterization of swelling of weakly charged hydrogels subjected to amphiphile and cyclodextrin exposure. J. Colloid Interface Sci..

[29] Tierney S., Hjelme D.R., Stokke B.T. (2008). Determination of swelling of responsive gels with nanometer resolution. Fiber-optic based platform for hydrogels as signal transducers. Anal. Chem..

[30] Zhao B., Moore J.S. (2001). Fast pH- and ionic strength-responsive hydrogels in microchannels. Langmuir.

[31] Bouklas N., Huang R. (2012). Swelling kinetics of polymer gels: Comparison of linear and nonlinear theories. Soft Matter.

[32] Reynaldo L.P., Vologodskii A.V., Neri B.P., Lyamichev V.I. (2000). The kinetics of oligonucleotide replacements. J. Mol. Biol..

[33] Li Q.Q., Luan G.Y., Guo Q.P., Liang J.X. (2002). A new class of homogeneous nucleic acid probes based on specific displacement hybridization. Nucleic Acids Res..

[34] Srinivas N., Ouldridge T.E., Sulc P., Schaeffer J.M., Yurke B., Louis A.A., Doye J.P.K., Winfree E. (2013). On the biophysics and kinetics of toehold-mediated DNA strand displacement. Nucleic Acids Res..

[35] Livshits M.A., Mirzabekov A.D. (1996). Theoretical analysis of the kinetics of DNA hybridization with gel-immobilized oligonucleotides. Biophys. J..

[36] Kaneko Y., Sakai K., Kikuchi A., Yoshida R., Sakurai Y., Okano T. (1995). Influence of freely mobile grafted chain length on dynamic properties of comb-type grafted poly(*N*-isopropylacrylamide) hydrogels. Macromolecules.

[37] Bin Imran A., Seki T., Takeoka Y. (2010). Recent advances in hydrogels in terms of fast stimuli responsiveness and superior mechanical performance. Polym. J..

[38] Annabi N., Nichol J.W., Zhong X., Ji C.D., Koshy S., Khademhosseini A., Dehghani F. (2010). Controlling the porosity and microarchitecture of hydrogels for tissue engineering. Tissue Eng. Part B Rev..

[39] Gao M., Toita S., Sawada S., Akiyoshi K., Stokke B.T. (2013). Cyclodextrin triggered dimensional changes of polysaccharide nanogel integrated hydrogels at nanometer resolution. Soft Matter.

[40] Prot V., Sveinsson H.M., Gawel K., Gao M., Skallerud B., Stokke B.T. (2013). Swelling of a hemi-ellipsoidal ionic hydrogel for determination of material properties of deposited thin polymer films: An inverse finite element approach. Soft Matter.

[41] Caykara T., Bulut M., Dilsiz N., Akyuz Y. (2006). Macroporous poly(acrylamide) hydrogels: Swelling and shrinking behaviors. J. Macromol. Sci. Pure Appl. Chem..

[42] Caykara T., Bulut M., Demirci S. (2007). Preparation of macroporous poly(acrylamide) hydrogels by radiation induced polymerization technique. Nucl. Instrum. Methods Phys. Res. Sect. B.

[43] Gemeinhart R.A., Chen J., Park H., Park K. (2000). pH-sensitivity of fast responsive superporous hydrogels. J. Biomater. Sci. Polym. Ed..

[44] Lin D.C., Yurke B., Langrana N.A. (2004). Mechanical properties of a reversible, DNA-crosslinked polyacrylamide hydrogel. J. Biomech. Eng. Trans. Asme.

